# Effect of pH on the Kinetics of Cysteine-to-Cystine Conversion in Thin Films Formed by ESI Microdroplet Deposition

**DOI:** 10.1021/jasms.5c00195

**Published:** 2025-08-29

**Authors:** Marta Managò, Chiara Salvitti, Anna Troiani, Alessia Di Noi, Andreina Ricci, Federico Pepi

**Affiliations:** † Department of Chemistry and Technology of Drugs, 9311 Sapienza University of Rome, P.le Aldo Moro 5, 00185 Rome, Italy; ‡ Department of Mathematics and Physics, University of Campania L. Vanvitelli, Viale Lincoln 5, 81100 Caserta, Italy

**Keywords:** ESI mass spectrometry, Microdroplets, Thin film, Cysteine, Cystine

## Abstract

The oxidation of cysteine to cystine was investigated in aqueous thin films generated by the deposition of electrospray ionization (ESI) microdroplets. The confined volume of the thin film promotes the reaction, resulting in up to 80% conversion of cysteine to cystine at a thin film temperature of 40 °C. The pH of the solution is a critical parameter, influencing both the yield and the kinetics of the reaction. Strong reaction acceleration factors in thin film with respect to the bulk were measured.

## Graphical abstract

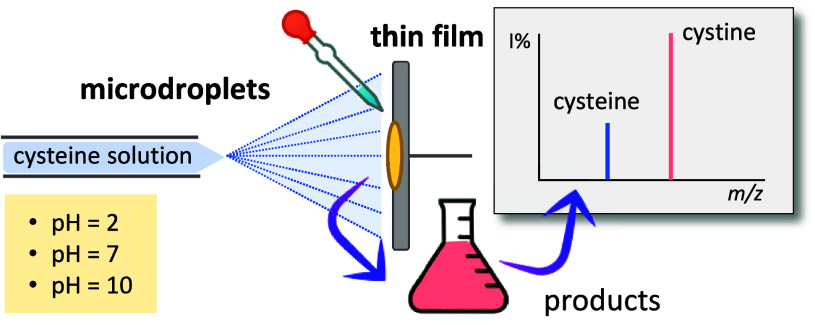

## Introduction

Cysteine plays a crucial role in cellular functions, primarily due to the redox activity, proton-donating capacity, and metal-binding properties of its reactive thiol group.^1^ The conversion of cysteine to cystine represents a key aspect of cellular redox regulation and occurs naturally in response to oxidative stress or alterations in the cellular environment (Scheme 1).^2^

**Scheme 1:**
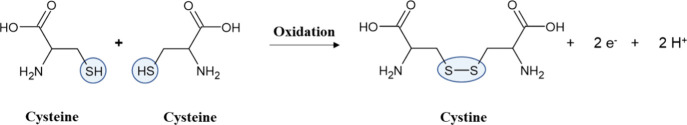
Cysteine Oxidation Reaction

This transformation is catalyzed by deprotonation of the sulfhydryl group to form the corresponding thiolate anion. In free cysteine, the p*K*_a_ of the thiol group is approximately 8.5, indicating that only a small fraction of cysteine exists in the deprotonated form at physiological pH.^3^ However, in proteins, the p*K*_a_ of cysteine residues can be significantly reduced due to electrostatic interactions with positively charged amino acids which stabilize the thiolate anion.^4,5^ Thus, the ionization and/or oxidation tendency of cysteine in aqueous solution is highly pH-dependent. In acidic conditions, cysteine remains predominantly in its less reactive, protonated thiol form, whereas under alkaline conditions the increased concentration of the thiolate anion (−S^–^) promotes its conversion to cystine.^6^

Cysteine oxidation in mammalian cells can be effectively modeled by studying this process in confined environments such as water microdroplets. The microdroplet reaction has been investigated under various experimental conditions in the presence of oxidizing agents such as ozone, hydrogen peroxide, hydroxyl radicals, or metal ions, which may be intrinsic to the droplet environment or externally introduced.^7–9^ In recent years, it has been demonstrated that numerous fundamental reactions proceed significantly faster in the microdroplets generated by the electrospray ionization (ESI) source of a mass spectrometer.^10–20^ This acceleration has attracted growing interest as it frequently deviates from what is observed in solution or gas-phase environments. In particular, gas-phase ion–molecule reactions—extensively characterized for their rapid kinetics and simplified reaction pathways under low-pressure conditions—have long served as a benchmark for studying chemical reactivity in the absence of solvent.^21,22^ The unique environment of ESI microdroplets, however, introduces additional factors, such as high surface-to-volume ratios and extreme local concentrations, which can lead to markedly different reaction outcomes. These include also the stabilization of short-lived intermediates, the facilitation of atypical chemical transformations not observed in the bulk, and the possible breakdown of atmospheric molecules.^23–29^ Spontaneous oxidation of cysteine and other low-molecular-weight thiols under ambient conditions has been reported in both ESI-generated microdroplets and sea spray aerosol particles.^30,31^ The mechanism proposed is initiated by the formation of the thiolate anion, which is particularly susceptible to oxidation by strongly oxidizing species, such as hydroxyl radicals. The interaction between the thiolate anion and the •OH radical generates an RS• radical that can rapidly dimerize, forming a disulfide bond, thereby yielding cystine. In pure water, only cystine formation has been observed with a conversion efficiency of approximately 7%.^30^ Conversely, in the presence of salts and in the absence of a strong electric field, additional oxidation products, such as sulfenic, sulfinic, and sulfonic acid derivatives, are also formed.^31^

Under appropriate conditions, such as those provided by an ESI Z-spray source, the microdroplets generated during electrospray ionization—which travel orthogonally to the mass spectrometer inlet—can be collected onto a target surface, forming a stable thin film characterized by a high surface-to-volume ratio that may promote the same reactions observed in microdroplets.^32^ Conducting reactions in thin films not only enables the isolation and quantification of neutral products—analyzed using the same mass spectrometer—but also extends the technique’s applicability to preparative synthetic purposes.^33–42^ Additionally, this experimental setup permits the discrimination of contributions from the microdroplet and thin film environments to product formation. Notably, the thin film retains the confined volume characteristic of microdroplets, which is essential for reaction acceleration, while allowing the reaction lifetime to be extended to longer time scales. This makes it possible to perform comprehensive kinetic analyses of the chemical transformations occurring within the film. The cysteine oxidation in thin films is particularly relevant to biointerfaces and sensor applications, where interactions with external stimuli influence its oxidation state and, consequently, the functional properties of the material. In biosensing and bioelectronics, cysteine-modified thin films are used for the detection of oxidative stress, environmental pollutants, and disease biomarkers.^43–46^

In this study, the cysteine-to-cystine conversion within thin films generated by deposition of negatively charged ESI microdroplets was investigated by using water solutions with different pH. Relative reaction rate constants were measured to assess the pH dependence of the reaction kinetics. Apparent acceleration factors for the thin film reaction were calculated by comparing the kinetic data obtained from the thin film experiments with those acquired for the same reaction performed in the bulk.

## Experimental Section

### Reagents

l-Cysteine (98%), l-cystine (98.5%), and all other chemicals were purchased from Sigma-Aldrich (St. Louis, MO, USA) and used without further purification. All solutions and dilutions were prepared exclusively with ultrapure water, and no organic solvents were introduced at any stage of the procedure.

### Mass Spectrometric Experiments

Microdroplet deposition experiments were conducted using the Z-spray electrospray ionization (ESI) source of a quadrupole time-of-flight (Q-TOF) mass spectrometer (Ultima, Waters, Manchester, UK), which was suitably modified for thin film reaction studies.^32^ In the Z-configuration, charged microdroplets are generated in-line with the spray axis and can be directed toward a stainless-steel target positioned 2.8 cm from the capillary tip, while the ion beam is deflected by 90° into the mass spectrometer for mass analysis.

Starting solutions were freshly prepared in amber glass vials by dissolving an appropriate amount of cysteine in pure water to obtain a 400 μM concentration immediately prior to each experiment. The acidic (pH 2) and alkaline (pH 10) solutions were obtained by adjusting the pH of the starting solution (initial pH ≈ 7) with concentrated hydrochloric acid and ammonium hydroxide, respectively. These solutions were infused into the ESI source operating in negative-ion mode, and the ionic composition of the microdroplets was characterized before the initiating thin film deposition experiments. The ESI source and the syringe containing the solution were both shielded from light.

The ESI parameters were as follows: desolvation gas (nitrogen) flow rate, 200 L h^–1^; source temperature, 80 °C; desolvation temperature, 200 °C; capillary voltage, −2.0 kV; cone voltage, −120 V; and infusion flow rate, 20 μL min^–1^. Mass spectra were collected by averaging 100 scans over the 50–600 *m*/*z* range.

Following acquisition of the initial (zero-time) mass spectrum, 1 mL of each cysteine solution was infused into the ESI source, and the resulting charged microdroplets were collected onto a stainless-steel plate. The temperature of the plate was monitored by using a thermocouple and measured to be 40 °C.

Microdroplet deposition onto the target surface led to the formation of a thin film from which a solid residue precipitated following solvent evaporation. The precipitate was rinsed with the same solvent mixture used in the spraying process after complete drying of the thin film. This drying typically occurred within two min after the end of microdroplet deposition. The resulting solution was analyzed with the same ESI parameters applied for acquiring the zero-time spectra. A scheme of the experimental setup is reported in Figure S1.

Structural characterization of cysteine and cystine ions was performed by collision-induced dissociation (CID) experiments. CID mass spectra were obtained by introducing argon as the collision gas into the quadrupole collision cell at a pressure of 0.1–0.5 mTorr.

### Yield Estimation

Rough estimates of the reaction yield in the thin film were obtained by calculating the conversion ratio (CR), defined as [P]/[P]+[R], where [P] and [R] are the signal intensities of the product and reactant ions in the mass spectrum, respectively. Three independent measurements were performed on different days by different operators and averaged together. Absolute yields were determined by correcting the conversion ratios for an ionization factor. For this purpose, standard solutions with different known molar ratios of cysteine and cystine at pH 2, 7, and 10 were prepared and analyzed under identical ESI conditions. The ratio of the ion signal intensities was then plotted against the theoretical molar ratios to derive the ionization factors.

### Kinetic Measurements

The kinetics of cysteine oxidation in the thin film were studied by extending the thin film’s lifetime while maintaining a constant concentration of cysteine. This was achieved by initially depositing the cysteine solution for a sufficient time to accumulate an analyzable amount of the reactant without its complete conversion into the products. Subsequently, a pure solvent was infused to sustain the thin film volume over time. This method ensures continuous renewal of the solvent environment without altering the amount of reactant, thus enabling accurate kinetic measurements. Relative rate constants at different pH values were determined by comparing kinetic profiles within a self-consistent reaction model (vide infra). Analogous kinetic measurements were performed in bulk by stirring 50 mL of 1 mM cysteine solutions (pH 2, 7, and 10) at 40 °C. The reaction mixture was sampled at different time intervals and analyzed by ESI-MS under the same experimental conditions used for the thin film studies. To assess the reproducibility, all of the kinetic experiments were repeated three times on separate days.

### Apparent Acceleration Factor (AAF)

The apparent acceleration factor (AAF) of the reaction in the thin film was calculated by the ratio of the measured kinetic constant in the thin film to that obtained for the same reaction performed in bulk.

### Data Analysis

Mass spectrometric data acquisition and processing were performed using MassLynx software (ver. 4.0, Waters Corp.).

## Results and Discussion

In this study, the kinetics of cysteine oxidation to cystine in aqueous thin films formed by deposition of ESI-generated negatively charged microdroplets onto a stainless-steel surface was investigated at three different pH values: 2, 7, and 10. The mass spectra of the starting solutions, which reflect the ionic composition of the deposited microdroplets, are shown in Figure 1. Under neutral and alkaline conditions, the spectra are dominated by two peaks at *m*/*z* 120 and *m*/*z* 241, corresponding to the deprotonated cysteine anion and its dimer, respectively (Figure 1a, b). In contrast, at pH 2, the acidic environment suppresses the formation of these deprotonated species; however, the mass spectrum still shows intense signals that can be attributed to cysteine, as evidenced by the formation of chloride adducts (Figure 1c). Prominent peaks appear at *m*/*z* 156 and 158, corresponding to the [Cysteine + ^35^Cl]^−^ anion and its ^37^Cl isotopic counterpart, respectively, along with minor signals at *m*/*z* 277 and 279, corresponding to chloride-bound cysteine dimers, [(Cysteine)_2_ + Cl]^−^.

**Figure 1:**
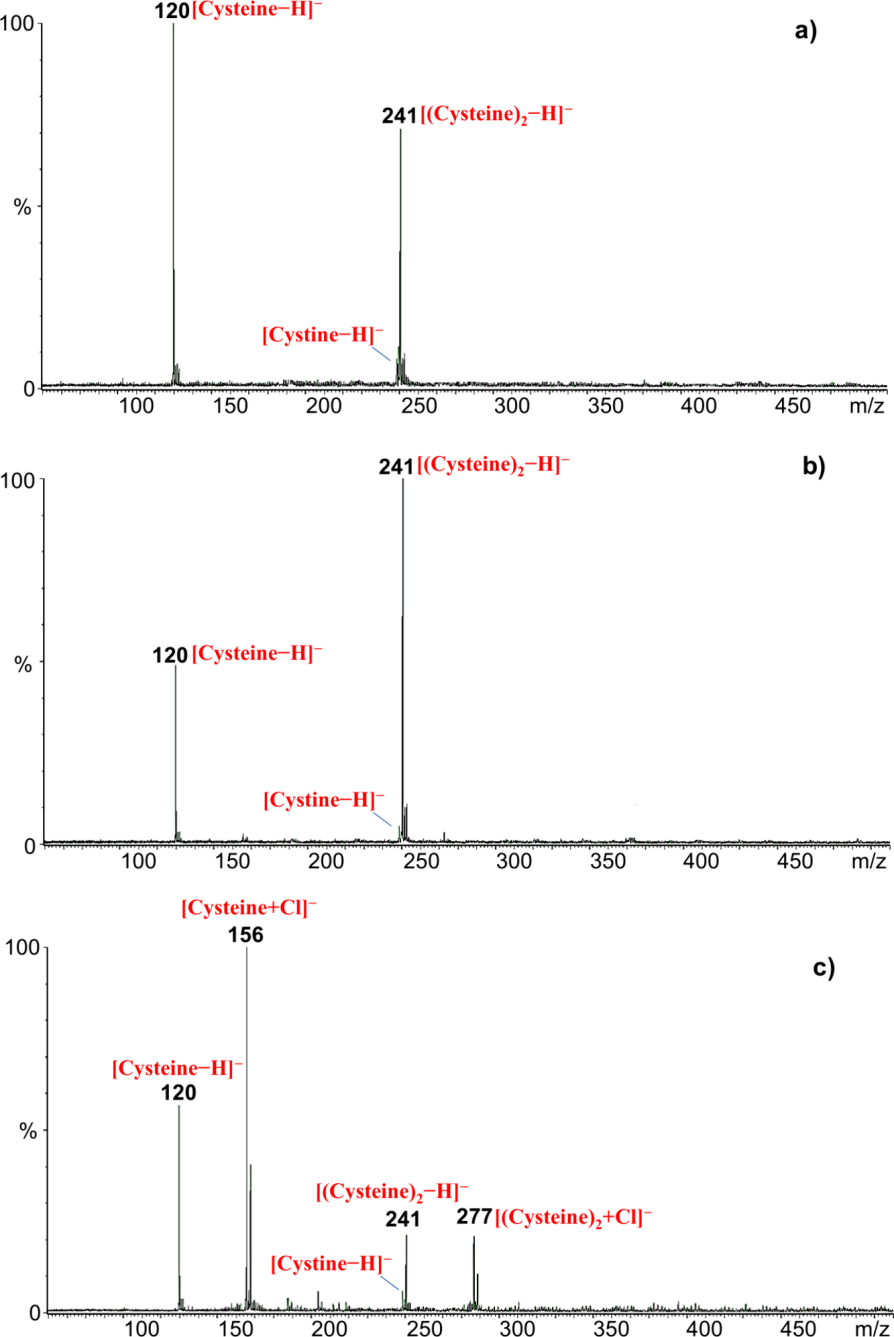
Negative ion mode ESI mass spectra of the 400 μM cysteine starting solutions at (a) pH 10, (b) pH 7, and (c) pH 2.

All freshly prepared solutions exhibit a minor signal at *m*/*z* 239, which can be attributed to the deprotonated cystine anion. This species likely forms in the microdroplets prior to their deposition. Its relative abundance, never exceeding 10% of the total ion signal, is consistent with previously reported values for cysteine oxidation occurring in negatively charged ESI microdroplets.^30^

Deposition of the aqueous microdroplets onto the stainless-steel target surface results in the formation of a stable thin film from which a white precipitate separates at the end of the reaction. The precipitate was rinsed with the same solvent mixture used for microdroplet generation and subsequently analyzed by ESI mass spectrometry under the same experimental parameters as those set for the starting solutions. The ESI mass spectra of the samples obtained after 15 min of microdroplet deposition are shown in Figure 2.

**Figure 2:**
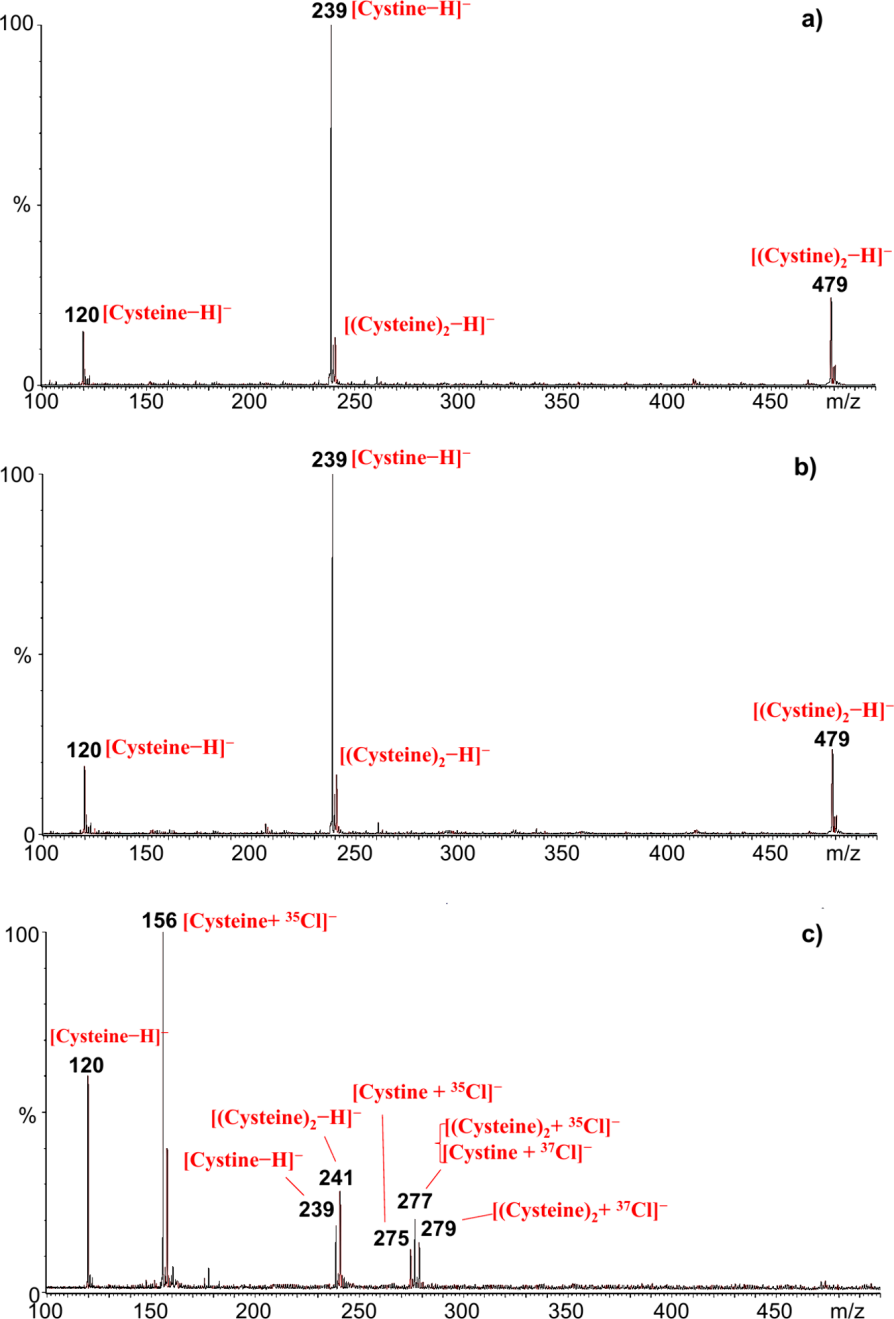
Negative ion mode ESI mass spectra of the rinsed precipitates formed after 15 min of microdroplet deposition at (a) pH 10, (b) pH 7, and (c) pH 2.

In addition to the anion at *m*/*z* 239, the formation of cystine is further supported by the presence of ionic species analogous to those associated with cysteine, namely the dimer anion at *m*/*z* 479 and, for the system at pH = 2, the chloride adducts at *m*/*z* 275 and *m*/*z* 277. No dimeric cystine Cl^–^ adducts at *m*/*z* 515 and *m*/*z* 517 were observed. It should be noted that the ion at *m*/*z* 277 is also one of the characteristic signals of cysteine, representing both the cysteine ^35^Cl^–^ bound dimer and the cystine adducts with ^37^Cl^–^.

Cysteine oxidation to cystine at pH 7 and 10 would appear to be clearly catalyzed by the confined environment of the thin film formed via microdroplet deposition, as evidenced by the intense signals at *m*/*z* 239 and *m*/*z* 479, which dominate over the characteristic cysteine peaks. As expected, the reaction is markedly less efficient at pH = 2, as shown by the relatively lower intensities of the cystine-related ions compared to those of cysteine. The mass assignments of all of the characteristic cysteine and cystine ions were confirmed via collision-induced dissociations (CID), as shown in Figures S2–S8. Fragmentations of the ion at *m*/*z* 277 confirmed that this signal results from the overlap of the above-described species. In this case, the intensity of each individual ion was determined by deconvoluting the isotopic pattern using the known relative abundances of the adjacent isotope peaks at *m*/*z* 275 and *m/*z 279.

In microdroplet thin film mass spectrometric studies, the conversion ratio (CR) is commonly used to estimate reaction yields. However, since CR is derived from the ratio of the ion intensities of reactants and products, it may be significantly affected by differences in ionization efficiency between the species. To correct for this, the relative ionization (RI) efficiencies of cysteine and cystine were determined by analyzing mixed solutions of the two compounds at varying molar ratios (RI_theor_). Calibration curves were constructed by plotting these theoretical ratios against the experimental ion intensity ratios (RI_exp_) obtained from the ESI mass spectra. The resulting plots (Figure S9) exhibited linear relationships with correlation coefficients (*R* ^2^) around 95%. Consistent with the presence of two ionizable carboxylic groups in cystine, the ionization correction factors were found to increase with pH, with values of 1.3 at pH 2, 1.6 at pH 7, and up to 1.8 under alkaline conditions. These correction factors were applied to calculate the reaction yields reported in Figure 3, also taking into account the stoichiometric 2:1 cysteine-to-cystine ratio. At pH 10, the yield of cystine reached a maximum of approximately 90% after only 10 min of microdroplet deposition (Figure 3a). Similar yields and deposition times were also observed under neutral pH conditions (Figure 3b), whereas the reaction conducted at pH 2 exhibited a maximum conversion of about ∼20%, even at extended deposition times (Figure 3c).

**Figure 3:**
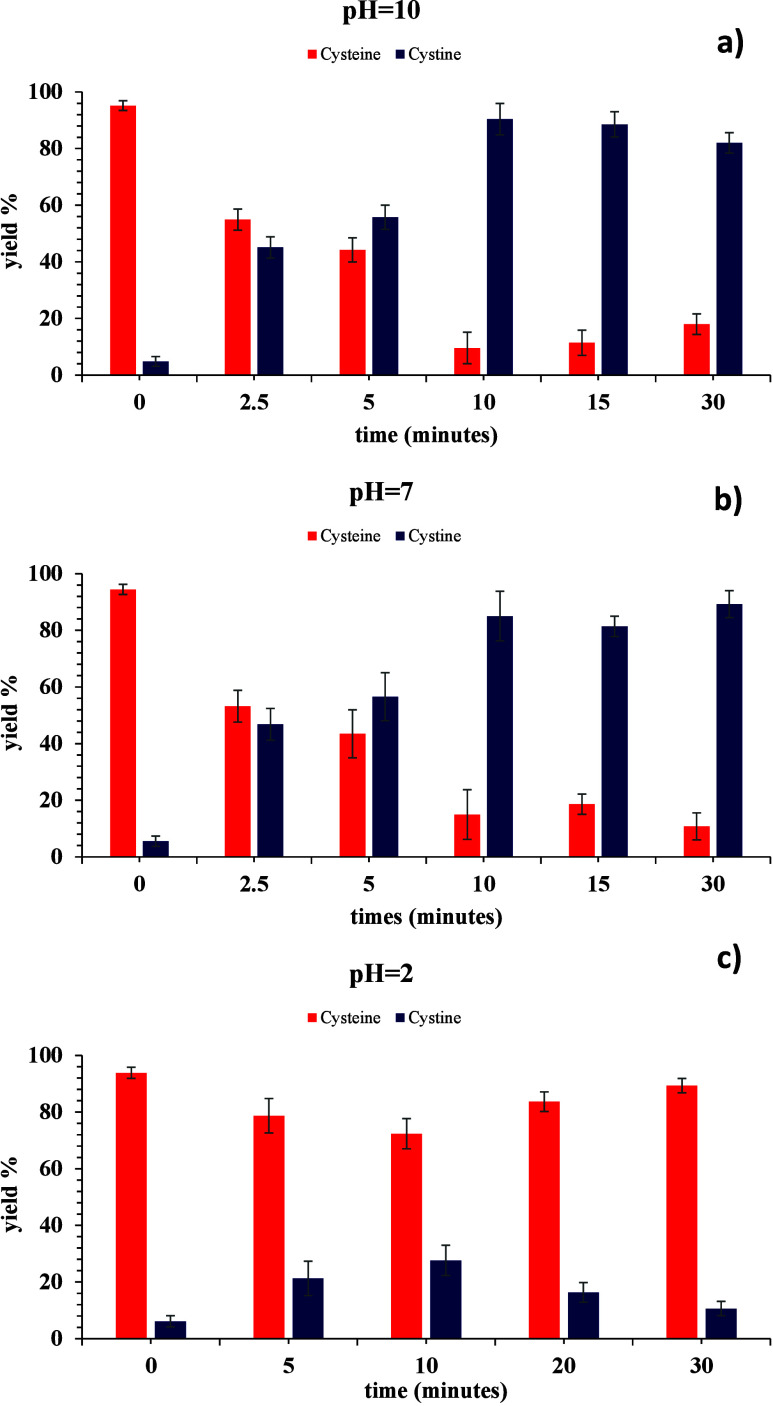
Corrected thin-film reaction yields at different microdroplet deposition times for the systems at (a) pH 10, (b) pH 7, and (c) pH 2. Error bars report the standard deviation (SD) obtained by averaging three independent measurements.

Under all tested pH conditions, the reaction yields initially increased, reaching a maximum before exhibiting a slight decline over time. To assess whether this evidence could be attributed to the possible reverse hydrolysis of cystine into cysteine, the reactivity of cystine in thin films was investigated at the three pH values by depositing ESI microdroplets from 400 μM aqueous cystine solutions. For comparison purposes, the solutions of the rinsed precipitates were always analyzed in negative-ion mode. Analysis of the thin film reaction products revealed only trace amounts of cysteine, thereby excluding the possibility that cystine undergoes hydrolysis to cysteine under the investigated pH conditions (Figure S10). The observed steady state followed by a gradual decrease over time could be due to the continuous addition of cysteine, which progressively increases its concentration, leading to a slow decline in the maximum observed yields.

To investigate whether the cysteine-to-cystine conversion in thin films is affected by the ionization process of the charged microdroplets, the reaction was also performed using microdroplets generated under a higher negative potential (−4 kV), in positive-ion mode under an applied voltage of +4 kV, and in the absence of any applied voltage. The reaction yields obtained under these conditions were nearly identical to those observed under −2 kV negative-ion mode, suggesting that the electric field does not significantly affect the reactivity within the thin films resulting from microdroplets deposition (Figure S11). Despite this, all the subsequent experiments were conducted in negative-ion mode at −2 kV to allow direct comparison with previous studies on cysteine microdroplet chemistry and because this mode provided superior ionization efficiency for the reaction products across the pH values tested.

### Kinetic Study of Cysteine-to-Cystine Oxidation in Thin Films and in the Bulk

The kinetics of cysteine oxidation to cystine in thin films was investigated by prolonging the lifetime of the thin film formed via microdroplet deposition without continuous addition of the reactant. Specifically, a fixed amount of cysteine solution was deposited onto the stainless-steel target surface for a short, controlled period—5 min for solutions at pH 10 and pH 7 and 15 min for the solution at pH = 2—sufficient to accumulate a measurable amount of unreacted cysteine while minimizing its conversion to cystine. Following this initial deposition step, only the solvent at the appropriate pH value was infused at defined time intervals. This approach allowed for the continuous renewal of the thin film environment without alteration of the initially deposited amount of cysteine, thereby maintaining a constant initial reactant concentration and reaction volume throughout the experiment. Relative kinetics constants at different pH values were obtained by comparing the resulting kinetic profiles within a self-consistent reaction model. Assuming that the reactant concentration corresponds to the ion intensity values corrected for the ionization efficiency and that cysteine oxidation follows a pseudo-first-order kinetics, the natural logarithm of the concentration ratio [cysteine]_*t*_/[cysteine]_*t*=0_ was plotted as a function of time. The resulting best-fit lines are shown in Figure 4.

**Figure 4:**
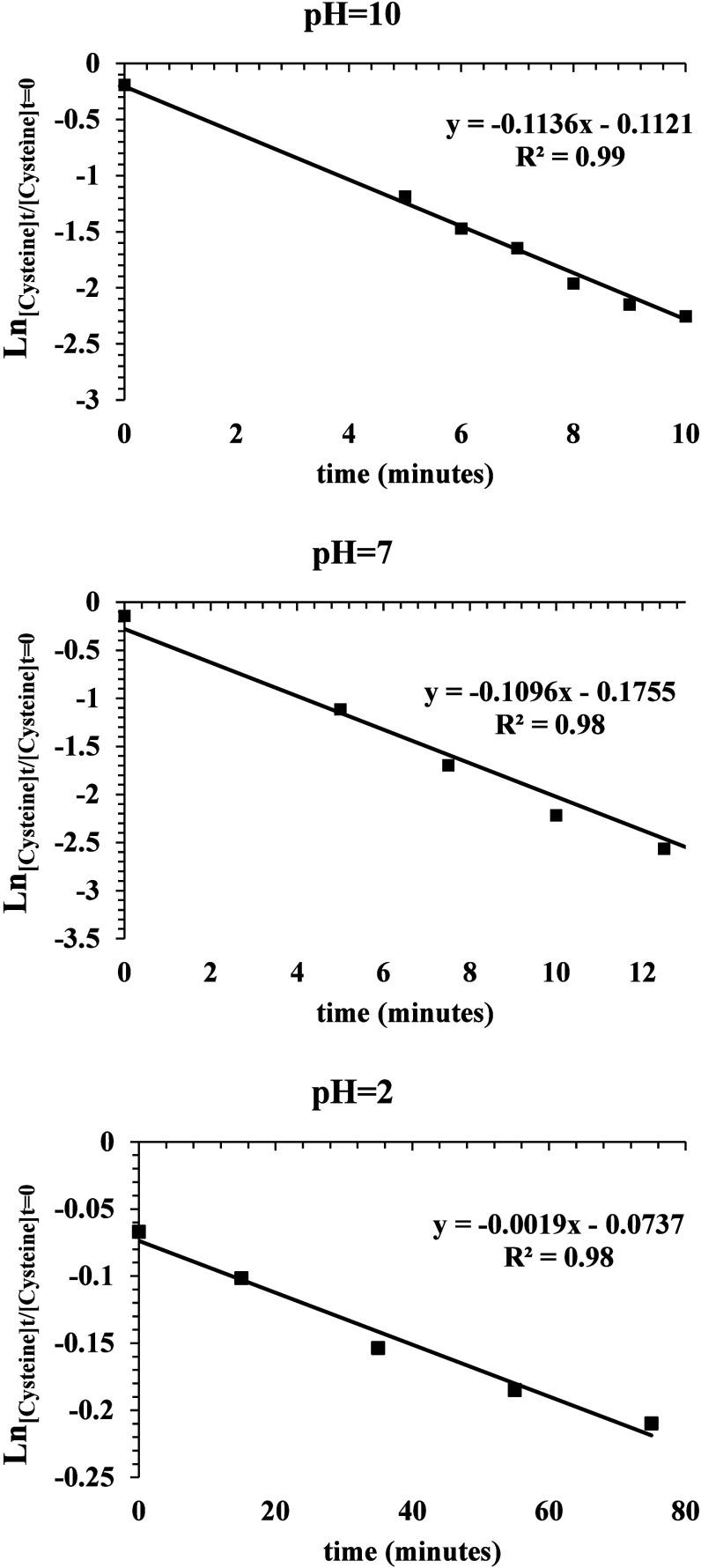
Kinetics plots of the thin film reactions at pH 10, 7, and 2. Time 0 corresponds to the microdroplet reactions, whereas 5 min for pH 10 and pH 7 and 15 min for pH 2 represent the starting points of the pure thin film reactions.

The slopes of the best-fit lines were used to determine the relative rate constants for cysteine oxidation in the thin film at different pH values. The initial points of the kinetic plots do not correspond to zero, as the mass spectra of the freshly prepared solutions already display ionic signals attributable to cysteine formed in the ESI microdroplets. The data at 5 min for the alkaline and neutral solutions and those at 15 min for the acidic solution represent the starting point of the pure thin film reaction—defined as the time at which only solvent microdroplets at the desired pH were delivered to the surface. During this initial interval, the reaction kinetics may still be influenced by the continued deposition of the reactant. However, as evident from all three kinetic profiles, these early data points are consistent with the trends observed at longer times and were therefore included in the linear regression analysis. The slopes of the regression lines for pH 10 and pH 7 are similar and approximately 2 orders of magnitude greater than the slope observed at pH 2 (Table 1). Cysteine oxidation in the thin film is slightly faster at pH 10 than at pH 7, with both reactions reaching a maximum yield after 10 and 12 min, respectively. In contrast, the reaction at pH 2 proceeds much more slowly, requiring at least 80 min to achieve its maximum conversion yield of ∼20%.

**Table 1:** Kinetic Parameters and Derived Apparent Acceleration Factors (AAFs) for the Investigation of Thin Film and Bulk Reactions

pH	Slope (Thin film)	Std Dev	Slope (Bulk)	Std Dev	AAF
10	0.1136	0.01	0.0014	0.00015	81.1
7	0.1096	0.04	0.0007	0.00013	147.5
2	0.0019	0.0003	0.0001	0.000009	19

To determine the apparent acceleration factors (AAFs) associated with cysteine oxidation in thin films, the same reaction was carried out in bulk under comparable experimental conditions (40 °C, 1 × 10^–3^ M cysteine). The kinetic plots for the bulk reactions are shown in Figure S12, while the slopes derived from the best-fit lines and their ratios relative to the thin film reactions are summarized in Table 1. Among the tested conditions, the thin film reaction at neutral pH and physiological temperature exhibited the highest acceleration factor: the conversion of cysteine to cystine occurred over ca. 150 times faster than that in solution. A slightly lower acceleration factor (∼80) was observed under alkaline conditions, likely due to the intrinsically faster oxidation kinetics of cysteine in the bulk at high pH values. Acidic cysteine solutions are generally considered stable, and in accordance with this, the oxidation reaction in bulk at pH 2 proceeded very slowly. Nonetheless, the thin film reaction under acidic conditions still proceeded approximately 20 times faster than the corresponding bulk process despite being significantly slower than the reactions at neutral or alkaline pH values. The reaction mechanism in thin films appears to mirror that of the bulk phase, wherein the formation of the thiolate anion constitutes a key step in the oxidation process. The thiolate anion predominates in alkaline solutions; moreover, the redox potential of the cysteine/cystine couple significantly decreases with increasing pH. The observed reaction acceleration, even in microdroplets generated without an applied potential, can be attributed to the increased reactant concentration resulting from solvent evaporation. The involvement of hydroxyl radicals (•OH)—commonly proposed in ESI microdroplet-induced cysteine oxidation—in the thin film reaction mechanism remains speculative, as no sulfonic or sulfenic acid derivatives were detected, nor were any cysteine–OH adducts observed in the mass spectra of the starting solutions.

## Conclusions

This study demonstrates that the oxidation of cysteine to cystine is significantly accelerated in thin films generated by the deposition of microdroplets with reaction kinetics strongly dependent on the pH of the starting aqueous solutions. Kinetic analysis revealed that the relative rate constants are approximately 2 orders of magnitude higher at neutral and alkaline pH compared to acidic pH. Comparison with corresponding bulk reactions indicates pronounced acceleration effects within the thin film environment, particularly at pH 7. Mechanistically, the formation of the deprotonated thiol group appears to be essential for promoting disulfide bond formation in the thin film. The enhanced reactivity can be attributed to the increased local concentration of reactants due to solvent evaporation, which also induces substantial shifts of pH relative to that of the original bulk solution. No evidence was found for the involvement of hydroxyl radicals or other highly oxidative species in the reaction pathway under the conditions employed. From this point of view, the strong oxidative effects observed in traditional in-line ESI sources seem to be partially suppressed in the Z-spray ESI source due to its orthogonal microdroplet trajectory relative to the mass spectrometer inlet. Conversely, the longer microdroplet path enhances desolvation, thereby increasing ionic concentrations in the thin film formed by their deposition onto the target solid surface. These findings provide insight into thin film chemistry and support its potential application in modeling reactive processes in confined aqueous environments, which may be useful in the development of cysteine-based sensing platforms.

## Supplementary Material



## Notes

Scheme of the experimental setup, CID mass spectra of the cysteine/cystine oxidation reaction products, calibration curves constructed to measure the ionization correction factors between cysteine and cystine at different pH values, ESI mass spectra of the standard cystine recovered from the thin film reaction at different pH values, effect of the capillary voltage on the thin-film reaction yields, and kinetic plots of the cysteine–cystine oxidation reaction performed in the bulk (PDF)
