# Supplementary material for: Effect of pH on the Kinetics of Cysteine-to-Cystine Conversion in Thin Films Formed by ESI Microdroplet Deposition
Source: J Am Soc Mass Spectrom. 2025 Aug 29;36(10):2189–96. doi: 10.1021/jasms.5c00195 (PMC12492406; doi:10.1021/jasms.5c00195)

Supporting Information

Effect of pH on the Kinetics of Cysteine-to-Cystine Conversion in Thin Films Formed by ESI Microdroplets Deposition

Marta Managò[a], Chiara Salvitti[a], Anna Troiani[a], Alessia Di Noi[a], Andreina Ricci[b] and Federico Pepi[a]*.

[a] "Sapienza" University of Rome, Department of Chemistry and Technology of Drugs, P.le Aldo Moro 5, 00185 Rome, Italy

[b] Department of Mathematics and Physics, University of Campania L. Vanvitelli, Viale Lincoln 5, 81100, Caserta, Italy.

Corresponding author: federico.pepi@uniroma1.it

Figure S1: Scheme of the experimental setup.

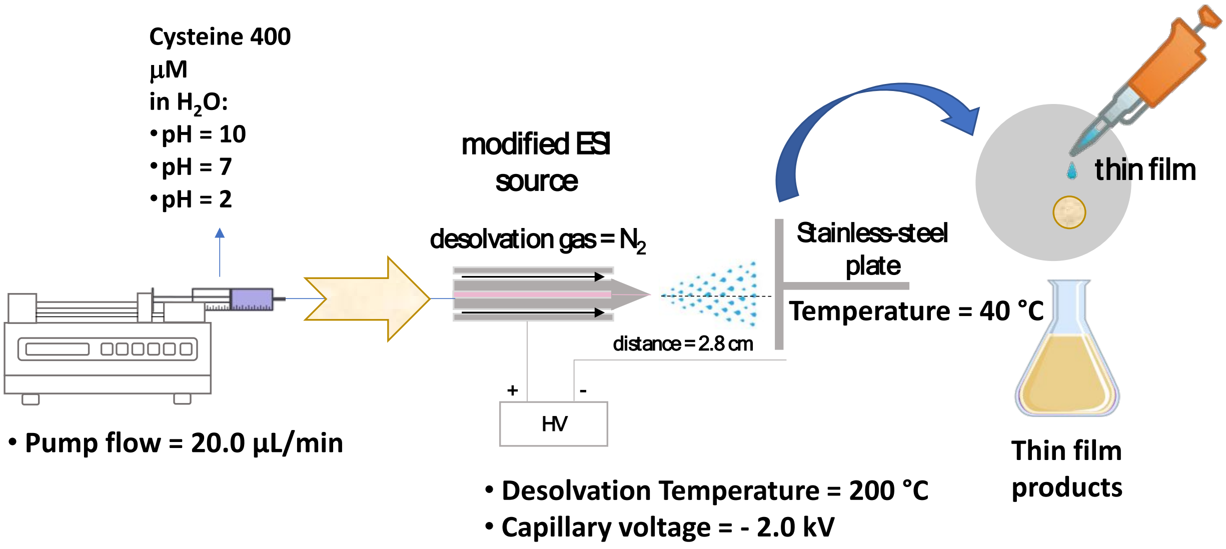

Figure S2: CID mass spectrum of [(Cysteine)$_2$-H]$^-$ at *m/z* 241

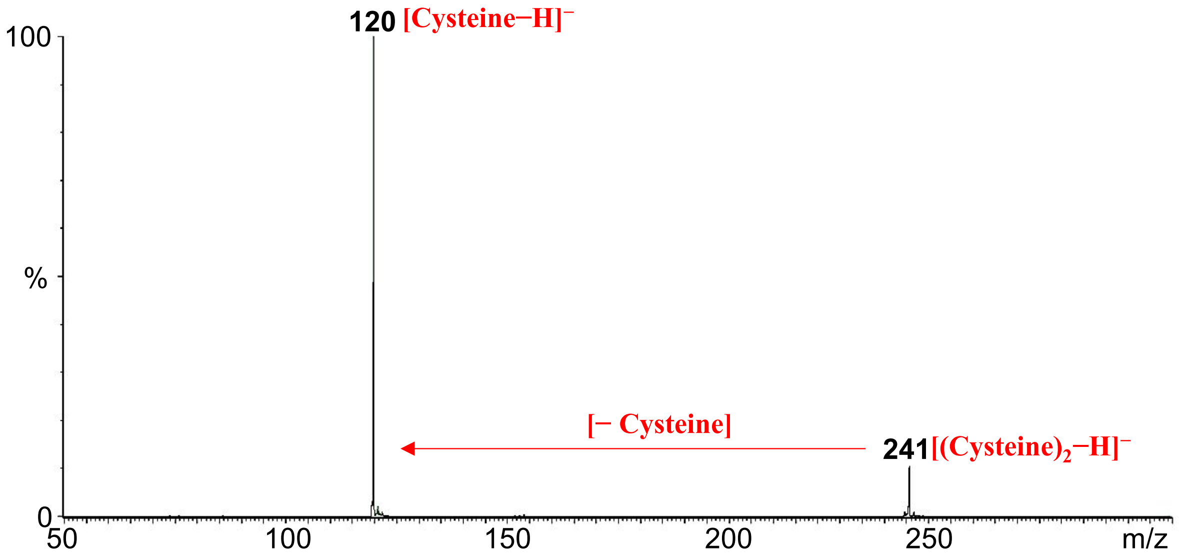

Figure S3: CID mass spectrum of [(Cystine)$_2$-H]$^-$ at *m/z* 479

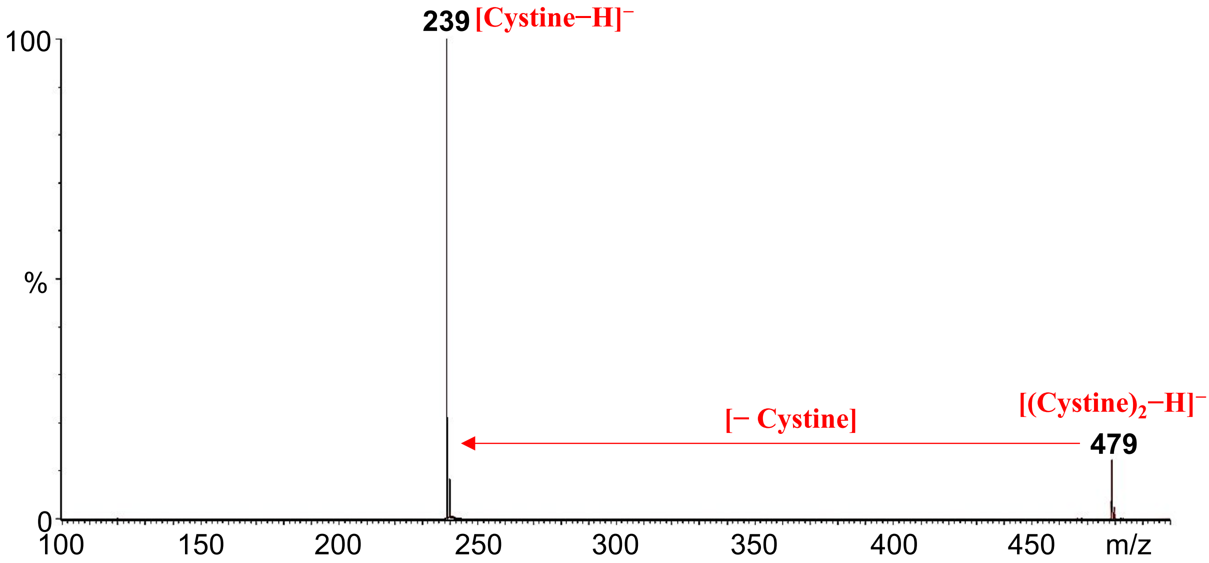

Figure S4: CID mass spectrum of [Cysteine + ^{35}Cl]$^{-}$ at *m/z* 156

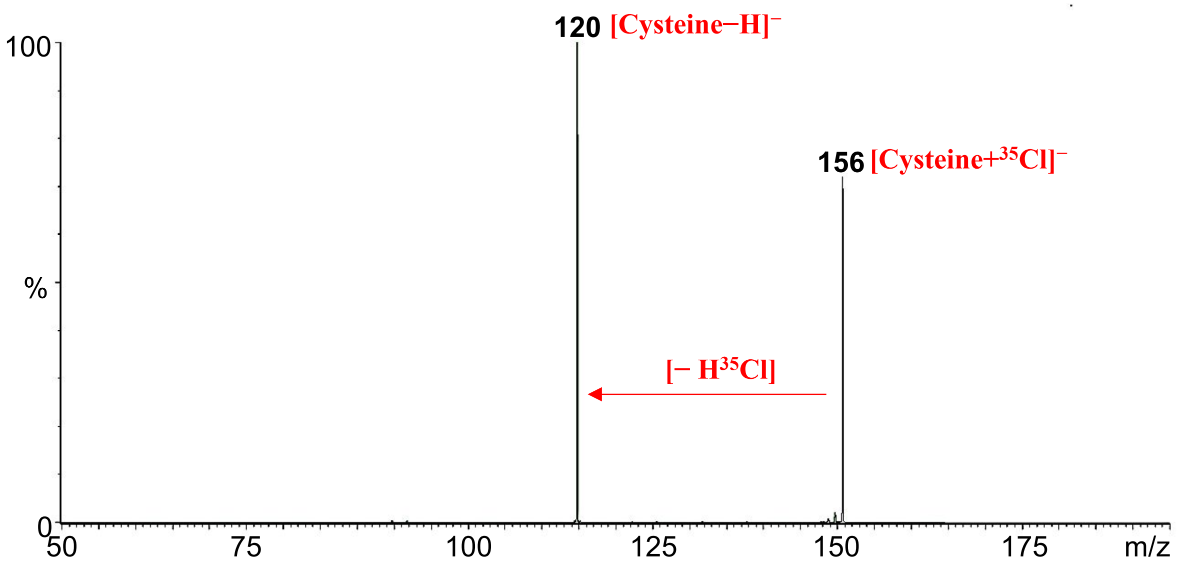

Figure S5: CID mass spectrum of [Cysteine + ^{37}Cl]$^{-}$ at *m/z* 158

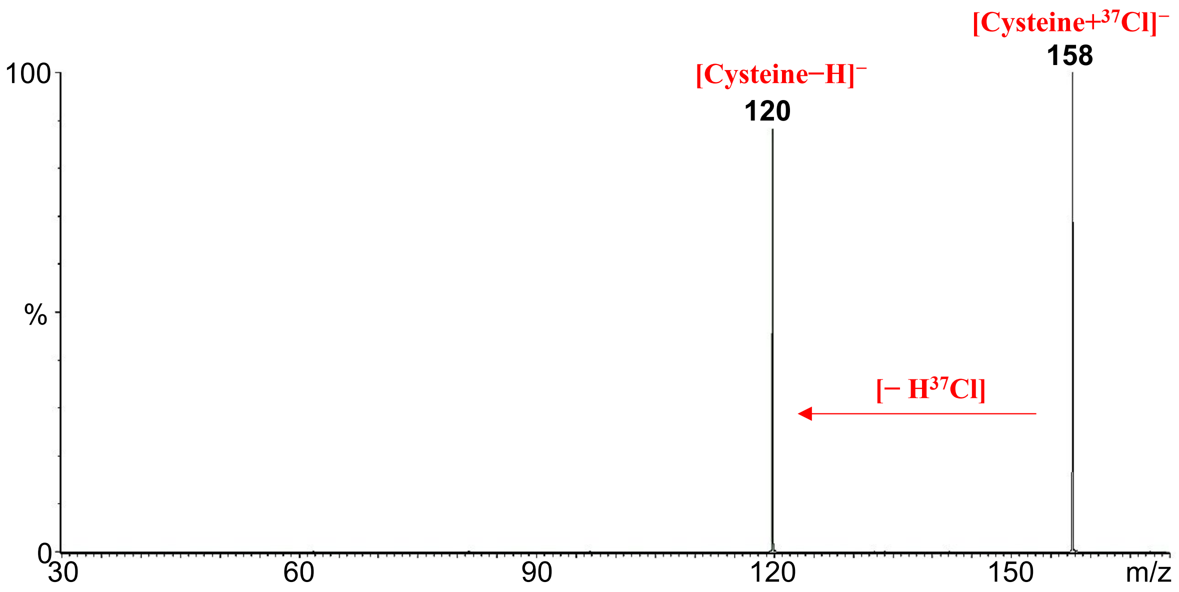

Figure S6: CID mass spectrum of [Cystine + ^{35}Cl]$^{-}$ at *m/z* 275

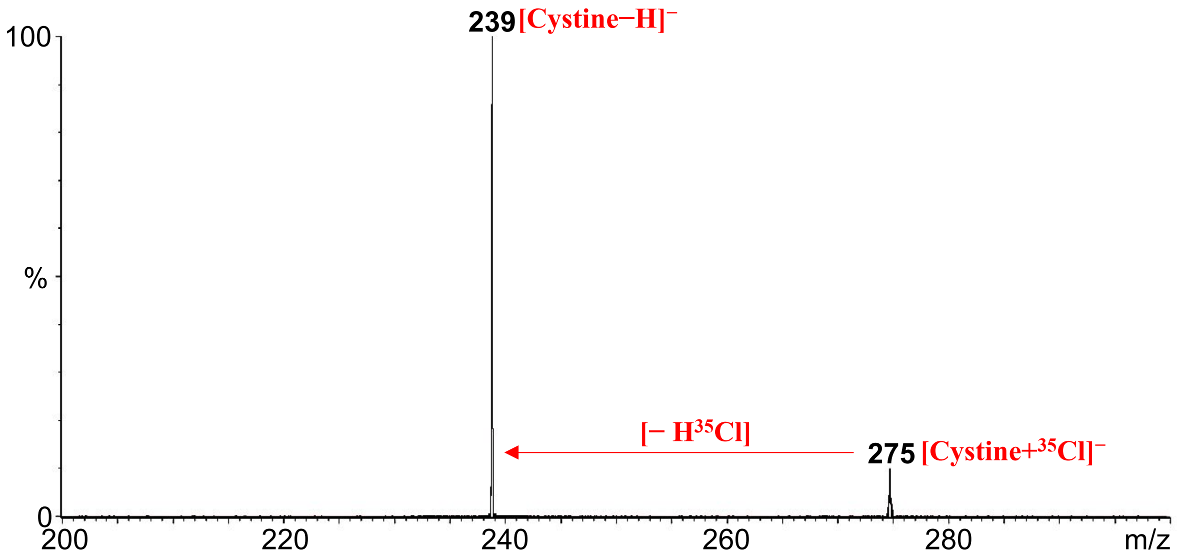

Figure S7: CID mass spectrum of [(Cysteine)$_2$ + ^{35}Cl]$^{-}$ and [Cystine + ^{37}Cl]$^{-}$ at *m/z* 277

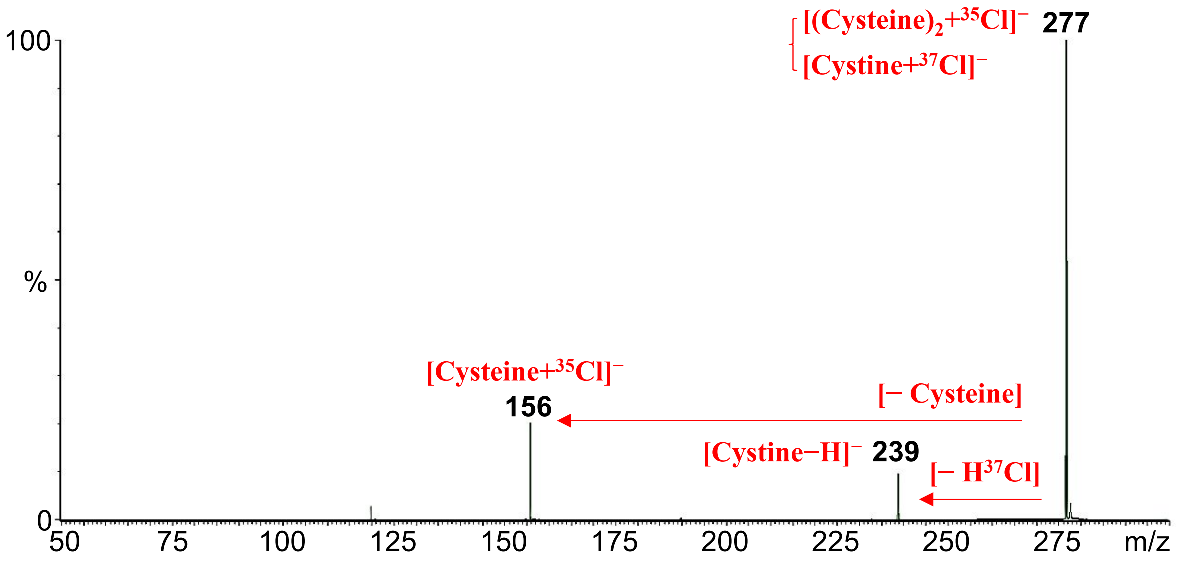

Figure S8: CID mass spectrum of $[(Cysteine)_2+^{37}Cl]^-$ at *m/z* 279.

The parent peak at *m/z* 279 contains also different combinations of S, Cl and C isotopes leading to the isotopic fragments at *m/z* 156, *m/z* 157 and *m/z* 243, namely: $[(Cysteine)(Cysteine^{34}S)+\ ^{35}Cl]^-$ and $[(Cysteine^{33}S)_2+\ ^{35}Cl]^-$/ $[(Cysteine^{33}S)(Cysteine^{13}C)+\ ^{35}Cl]^-$

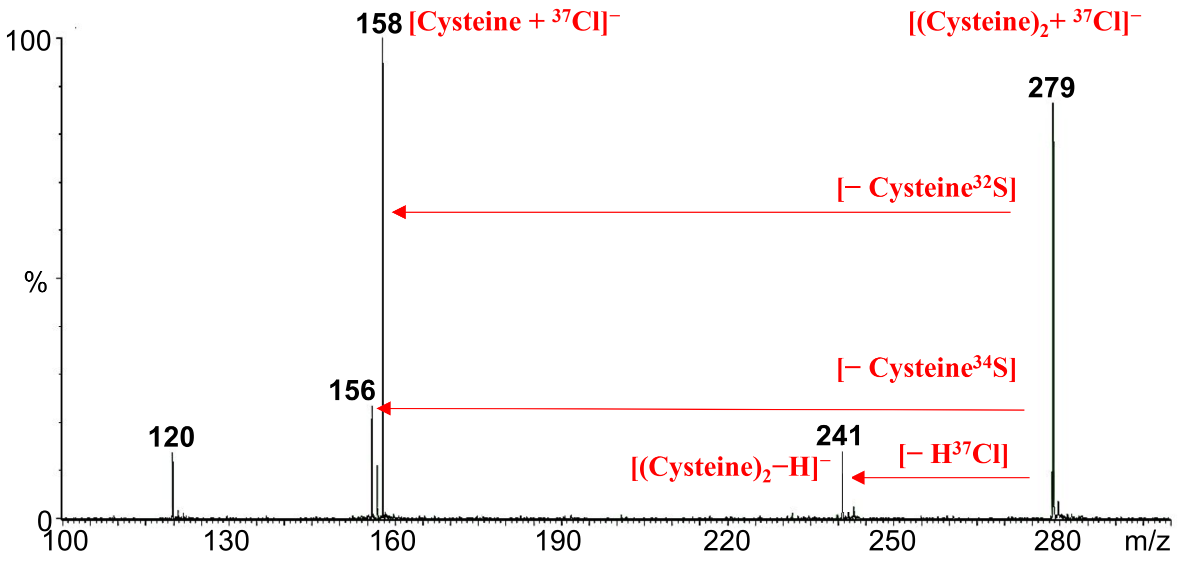

Figure S9: Ionization efficiency plots

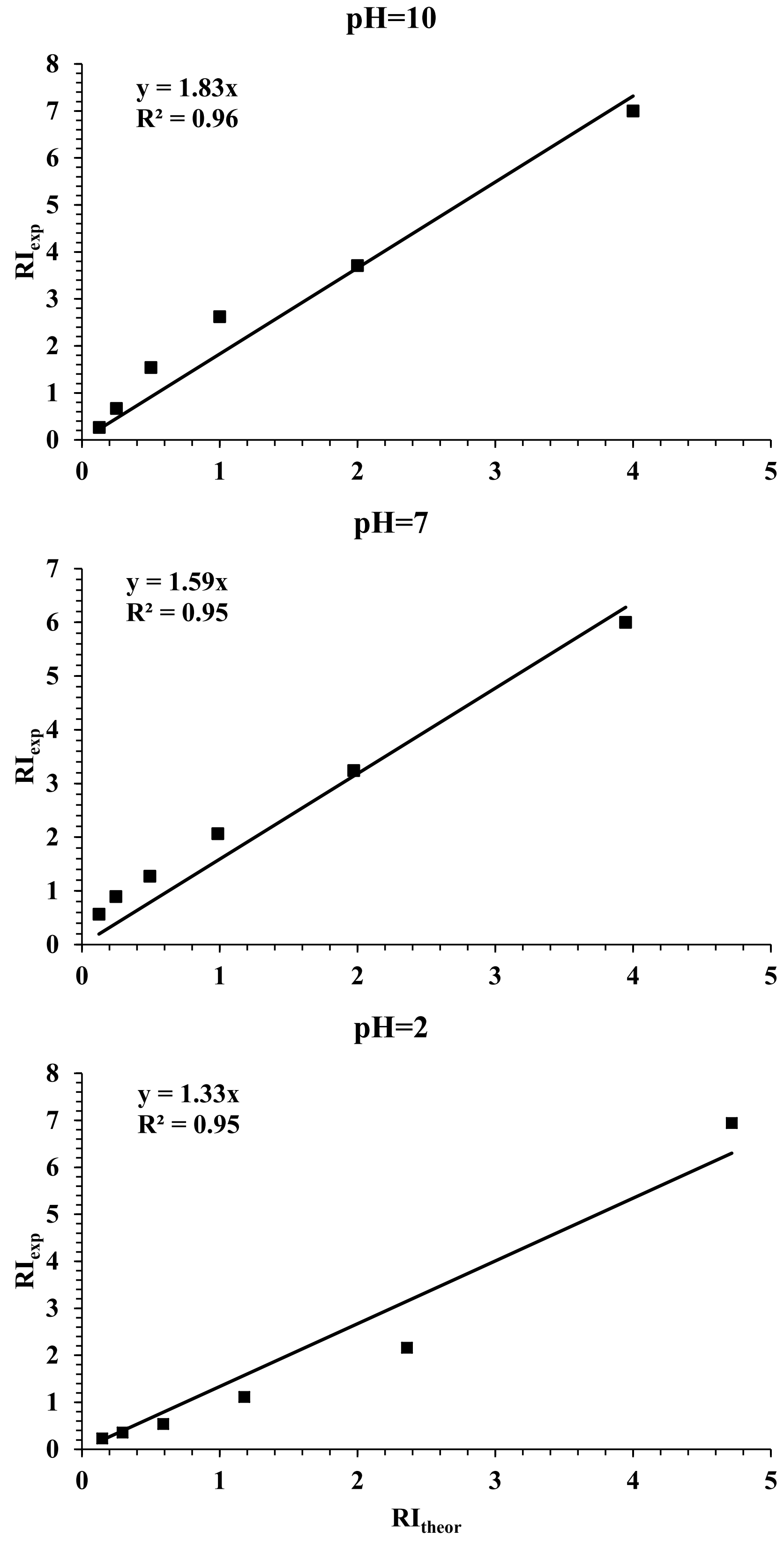

Figure S10: ESI mass spectrum of the thin film products obtained after 15 minutes of cystine 400 μM solution deposition at: a) pH=10, b) pH=7 and c) pH=2.

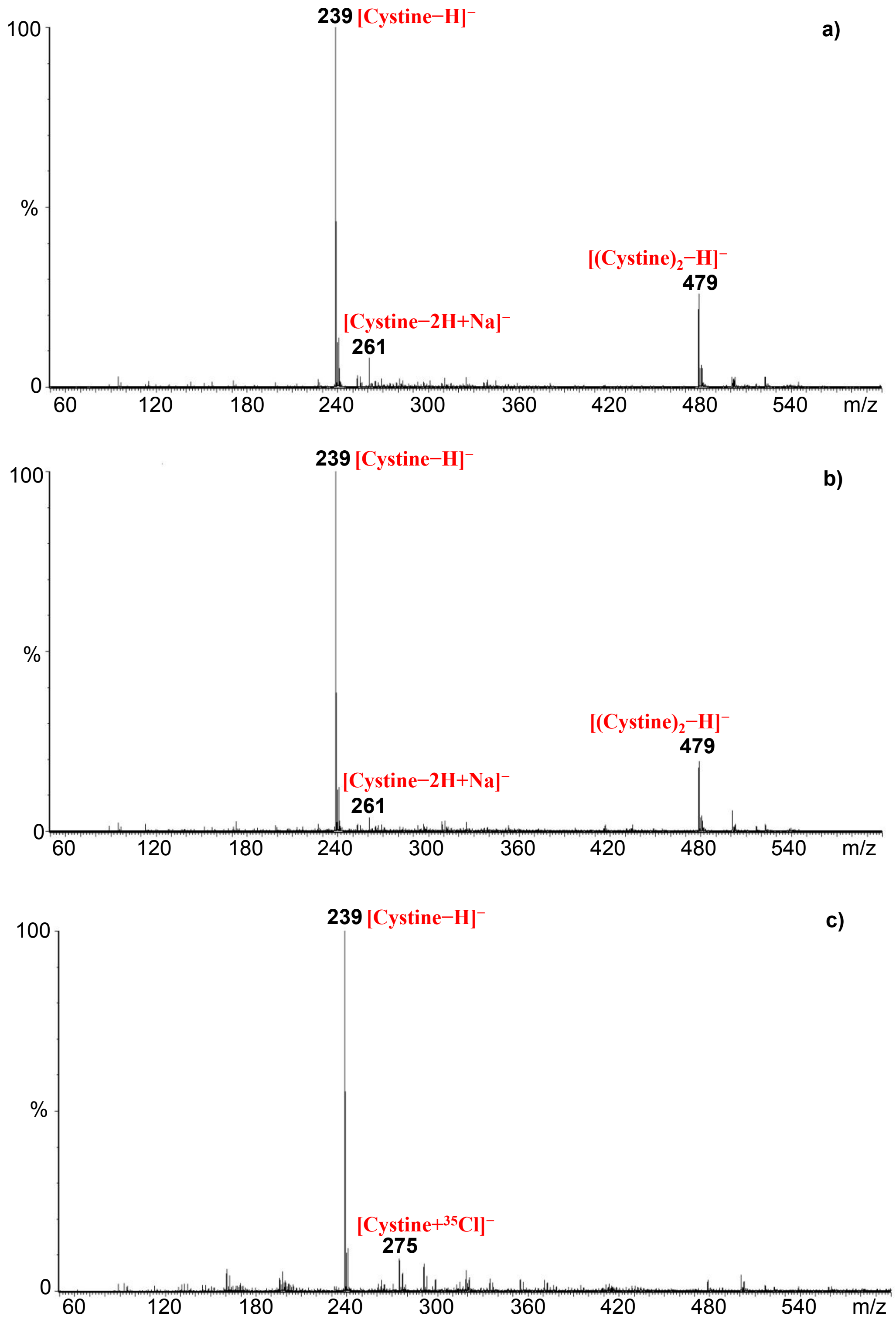

Figure S11: Effects of the ESI potential on the thin film reaction yields.

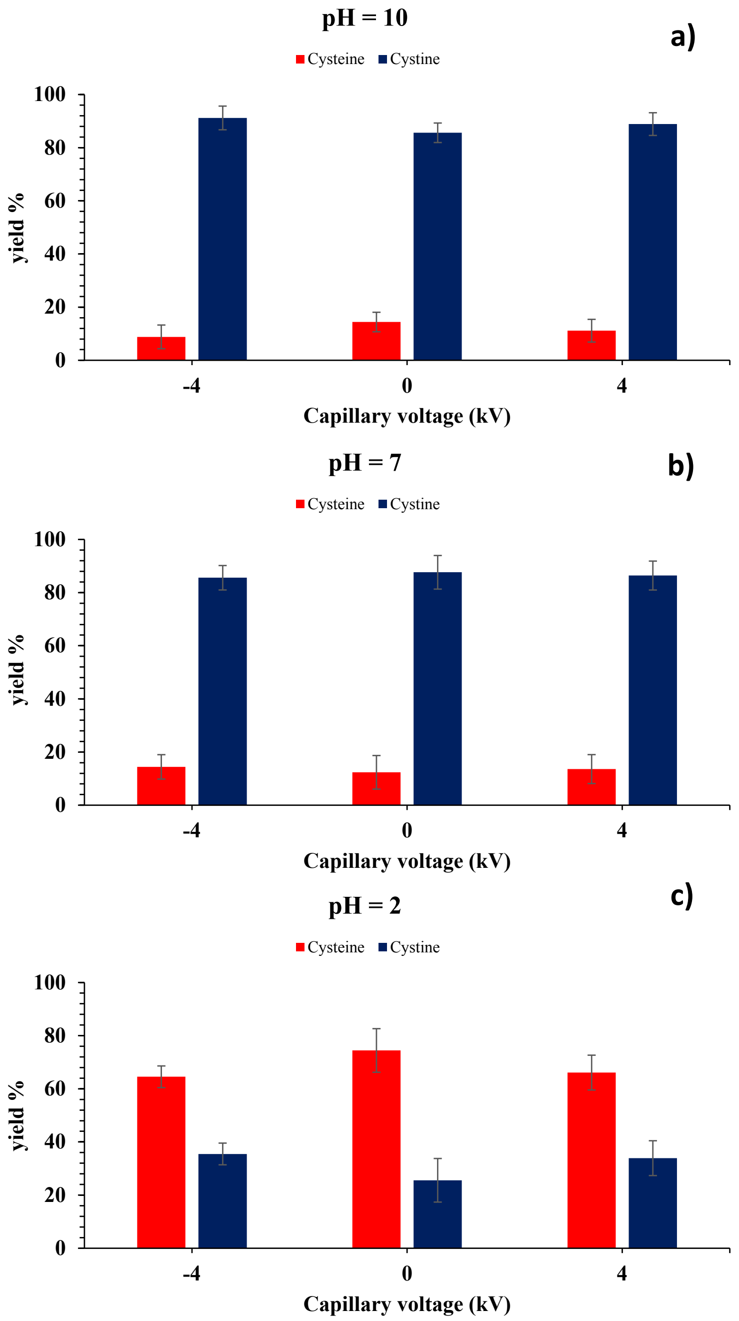

Figure S12: Kinetic plots of the bulk reactions.

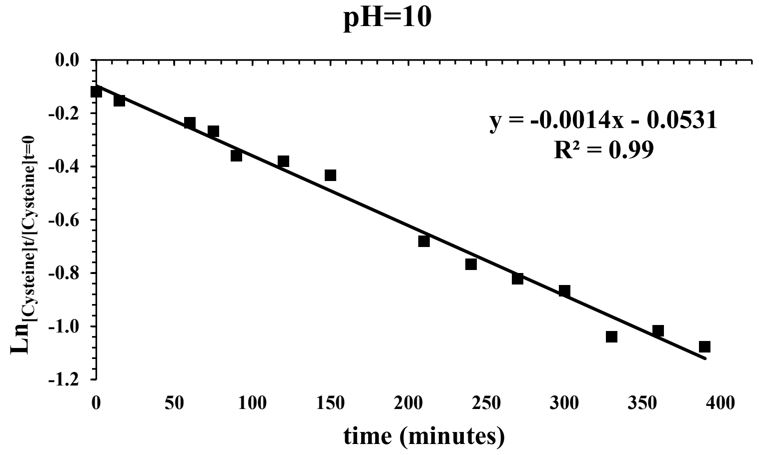

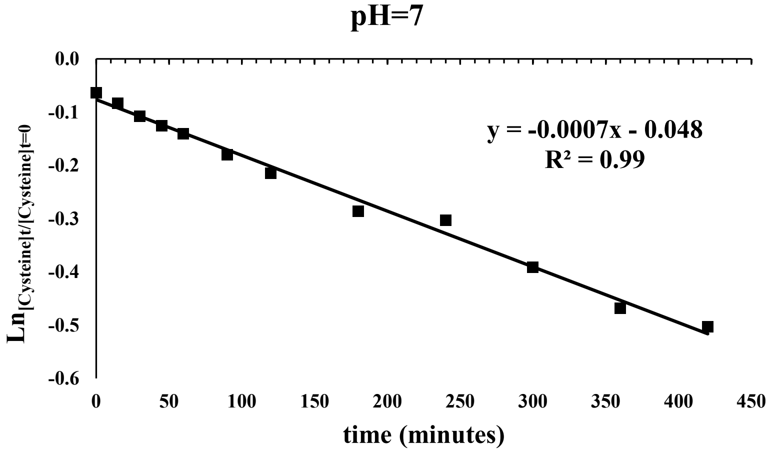

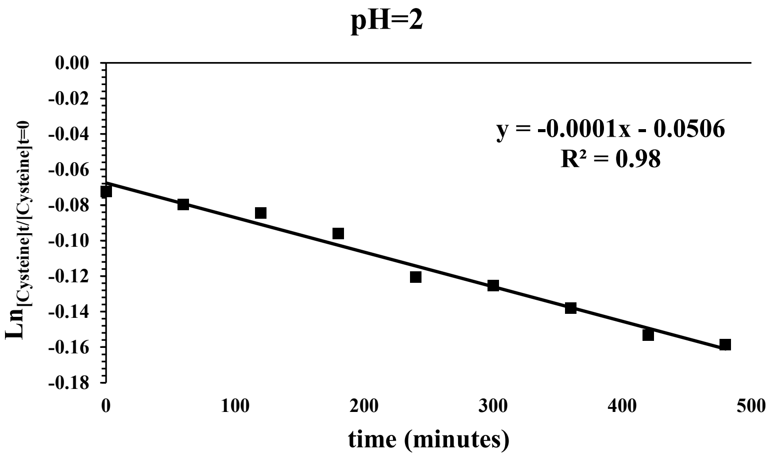

Supplement: Supplementary file 1 [file js5c00195_si_001.pdf]
